# Systolic Blood Pressure and 1-Year Clinical Outcomes in Patients Hospitalized for Heart Failure

**DOI:** 10.3389/fcvm.2022.877293

**Published:** 2022-04-25

**Authors:** Xinghe Huang, Jiamin Liu, Lihua Zhang, Bin Wang, Xueke Bai, Shuang Hu, Fengyu Miao, Aoxi Tian, Tingxuan Yang, Yan Li, Jing Li

**Affiliations:** ^1^Fuwai Hospital, Chinese Academy of Medical Sciences, Shenzhen, China; ^2^National Clinical Research Center for Cardiovascular Diseases, Fuwai Hospital, Chinese Academy of Medical Sciences and Peking Union Medical College, National Center for Cardiovascular Diseases, Beijing, China; ^3^School of Nursing, Chinese Academy of Medical Sciences and Peking Union Medical College, Beijing, China

**Keywords:** heart failure, hypertension, blood pressure, readmission, death

## Abstract

**Background:**

High systolic blood pressure (SBP) is an important risk factor for the progression of heart failure (HF); however, the association between SBP and prognosis among patients with established HF was uncertain. This study aimed to investigate the association between SBP and long-term clinical outcomes in patients hospitalized for HF.

**Methods:**

This study prospectively enrolled adult patients hospitalized for HF in 52 hospitals from 20 provinces in China. SBPs were measured in a stable condition judged by clinicians during hospitalization before discharge according to the standard research protocol. The primary outcomes included 1-year all-cause death and HF readmission. The multivariable Cox proportional hazards regression models were fitted to examine the association between SBP and clinical outcomes. Restricted cubic splines were used to examine the non-linear associations.

**Results:**

The 4,564 patients had a mean age of 65.3 ± 13.5 years and 37.9% were female. The average SBP was 123.2 ± 19.0 mmHg. One-year all-cause death and HF readmission were 16.9 and 32.7%, respectively. After adjustment, patients with SBP < 110 mmHg had a higher risk of all-cause death compared with those with SBP of 130–139 mmHg (HR 1.71; 95% CI: 1.32–2.20). Patients with SBP < 110 mmHg (HR 1.36; 95% CI: 1.14–1.64) and SBP ≥ 150 mmHg (HR 1.26; 95% CI: 1.01–1.58) had a higher risk of HF readmission, and the association between SBP and HF readmission followed a J-curve relationship with the nadir SBP around 130 mmHg. These associations were consistent regardless of age, sex, left ventricular ejection fraction, hypertension, coronary heart disease, and medications for HF.

**Conclusion:**

In patients hospitalized for HF, lower SBP in a stable phase during hospitalization portends an increased risk of 1-year death, and a J-curve association has been observed between SBP and 1-year HF readmission. These associations were consistent among clinically important subgroups.

## Introduction

Heart failure (HF) is a major global public health problem, with a prevalence of 64.3 million cases worldwide (1). HF is the leading cause of hospitalization among older patients and is associated with high mortality (2, 3). High blood pressure (BP) is a modifiable risk factor for incident HF, and clinical trials have demonstrated that lowering BP can substantially decrease the risk of developing HF (4–6). For patients with established HF, the latest American College of Cardiology/American Heart Association guidelines for HF and hypertension recommend that the optimal systolic blood pressure (SBP) in those with hypertension should be <130 mmHg; however, this recommendation is extrapolated from populations without HF (7, 8).

Among patients hospitalized for HF, previous studies have shown that those with lower admission SBP were at a higher risk of clinical outcomes (9–12); while the association between SBP during hospitalization and long-term clinical outcomes remains unclear (12–17). Some studies demonstrated that a low SBP level may have a paradoxical association with an increased risk of death (12, 13), and one study reported that a relatively normal SBP may also be associated with unfavorable outcomes (14); while others found a J-curve association between SBP and outcomes (16, 17). Although the prevalence of HF readmission is high, there is still a lack of knowledge about the association of SBP during hospitalization with long-term HF readmission after the index hospitalization and whether the association is causal or due to reverse causality (13, 14). Patients with HF represent a heterogeneous population and the association between SBP and clinical outcomes could be different among subgroups of clinical importance, such as the subgroups of left ventricular ejection fraction (LVEF), age, comorbidities, and medications (13, 18–20), which requires further investigation to help explore the causality.

Accordingly, this study aimed to investigate the association of SBP in a stable phase during hospitalization with 1-year clinical outcomes in the overall population and in clinically important subgroups based on a prospective multicenter cohort with a large number of patients hospitalized for HF, which could provide more evidence for future recommendations on BP management, and help physicians optimize BP management strategies to improve outcomes of patients with HF.

## Methods

### Study Design and Population

The study design of the China Patient-centered Evaluative Assessment of Cardiac Events Prospective Heart Failure Study (China PEACE 5p-HF Study) has been described previously in detail (21). In brief, we established a prospective, nationwide, multicenter cohort of acute HF involving 52 hospitals from 20 provinces, and this covers all the economic–geographic regions across the nation (Supplementary Material). Patients were identified as eligible if they were ≥18 years old, local residents, and hospitalized primarily because of the new-onset HF or decompensated chronic HF. From August 2016 to May 2018, patients were screened consecutively, and eligible ones were enrolled and signed the informed consent. Interviews were conducted to collect data from the enrolled patients during the index hospitalization as well as at 1 month, 6 months, and 1 year after discharge. Regarding the patients unable to attend in-person interviews, the trained investigators at the national coordinating center would conduct central telephone interviews.

The Central Ethics Committee at the Fuwai Hospital and Local Internal Ethics Committees at Study Hospitals have approved the China PEACE 5p-HF Study. The study was registered at www.clinicaltrials.gov (NCT02878811).

### Data Collection and Variables Definition

Clinical status, comorbidities, and medications were obtained *via* central medical record abstraction. We used a standardized questionnaire for information collection particularly regarding demographic characteristics, socioeconomic characteristics, smoking status, and self-reported health status by face-to-face interview during index hospitalization by trained local clinicians. The local clinicians entered data into laptop computers which were equipped with a customized electronic data collection system allowing real-time off-line logic checks to verify the accuracy and completeness of the data. The trained clinicians measured LVEF during hospitalization based on the standard echocardiogram protocol. Low-density lipoprotein cholesterol, N-terminal pro-brain natriuretic peptide (NT-proBNP), and serum creatinine were according to the central laboratory tests—blood and urine sample tests taken within 48 h of admission by unified protocol or the last local laboratory tests before discharge if the central laboratory tests were unavailable (missing rate < 2.8%).

The measurement of SBP was conducted on the upper arm of each participant in a stable phase during hospitalization before discharge [i.e., a period when a patient was in a stable condition during hospitalization judged by local clinicians; median: 7 days (interquartile range (IQR): 6–10)] using unified electronic BP monitor (Omron HEM-7111) by trained site investigators according to the standard research protocol. The mean SBP of two or three successive measurements was calculated (if the difference between the first and second measurement was >5 mmHg then taking the third measurement).

Patients were classified by LVEF into heart failure with reduced ejection fraction (HFrEF, defined as LVEF <40%), mid-range ejection fraction (HFmrEF, defined as LVEF 40–49%), and preserved ejection fraction (HFpEF, defined as LVEF ≥ 50%). HF types included decompensated chronic HF (defined as patients having had HF for a period of time and then being admitted because of the chronic stable HF deterioration) and new-onset HF. Hypertension, coronary heart disease, atrial fibrillation, diabetes, reduced renal function, anemia, stroke, chronic obstructive pulmonary disease, and valvular heart disease were defined according to the medical record (medical history or discharge diagnosis) or positive laboratory test results. We defined the diagnosis criteria of laboratory tests of anemia as hemoglobin <120 g/L in men or <110 g/L in women, reduced renal function as estimated glomerular filtration rate <60 ml/min/1.73 m^2^, and diabetes as HbA_1c_ ≥ 6.5%. And we evaluated self-reported health status using the short version of Kansas City Cardiomyopathy Questionnaire (KCCQ) sum score at baseline, the scores of which ranged from 0 to 100 (lower scores equal to poorer health status).

### Outcomes and Adjudication

The primary outcomes of this study were 1-year all-cause death and HF readmission. We also included cardiovascular death and all-cause readmission as secondary outcomes. Cardiovascular death was defined as sudden death, death due to HF, stroke, acute myocardial infarction, or other cardiovascular causes, or presumed/unknown cardiovascular death. Information on the clinical outcomes was collected from follow-up interviews and the national death cause database. We also collected medical records of potential outcome events to do central adjudication by trained clinicians at the national coordinating center.

### Statistical Analysis

Categorical variables were expressed as count and percentage and tested using the chi-square test; continuous variables were reported as mean ± SD or median (IQR) and tested by the one-way ANOVA or the Kruskal–Wallis test, as appropriate. For continuous variables, we performed tests for linear trend by entering the median value of each category of SBP as a continuous variable in the models; for categorical variables, a trend was tested with the Cochran–Armitage trend test (22). The probabilities of clinical outcomes were plotted using the cumulative incidence functions that accounted for the competing risks, and the differences between SBP groups were compared by Gray's test (23, 24). To quantify the associations between SBP (<110 mmHg, 110–119 mmHg, 120–129 mmHg, 130–139 mmHg, which was used as the reference, 140–149 mmHg, and ≥150 mmHg) and time to the occurrence of the clinical outcomes, Cox frailty models with random intercepts for hospitals were applied to calculate the unadjusted and adjusted hazard ratios (HRs) and to account for clustering within hospitals. For HF readmission and all-cause readmission, we performed Fine-Gray analyses with death as a competing risk; for cardiovascular death, non-cardiovascular death was considered as a competing risk (23). In the adjusted models, we adjusted for the following variables: demographics (age and sex), socioeconomic information (education level and marital status), clinical status [body mass index, heart rate at discharge, New York Heart Association (NYHA) functional class at discharge, LVEF type, and HF type], smoking status, comorbidities (hypertension, coronary heart disease, atrial fibrillation, diabetes, reduced renal function, anemia, stroke, chronic obstructive pulmonary disease, and valvular heart disease), laboratory test results (NT-proBNP and low-density lipoprotein cholesterol), medications during hospitalization and at discharge (angiotensin-converting enzyme inhibitors [ACEIs], angiotensin receptor blockers [ARBs], β-blockers, aldosterone antagonists, diuretics, digoxin, and nitrates), and KCCQ sum score. In addition, we used restricted cubic splines to examine the non-linear association between SBP and clinical outcomes with four knots; i.e., the 5, 35, 65, and 95th percentiles of SBP. Furthermore, we performed sensitivity analyses by excluding those who died within 30 days after discharge (*n* = 117) and those with SBP <90 mmHg (*n* = 98).

The interactions between SBP groups and subgroup parameters were also included in the Cox models when assessing the association of SBP with all-cause death and HF readmission. Subgroup parameters included age (<65 or ≥65 years), sex (male or female), LVEF type (HFrEF, HFmrEF, or HFpEF), HF type (decompensated chronic HF or new-onset HF), NT-proBNP (< median or ≥median), hypertension (yes or no), diabetes (yes or no), atrial fibrillation (yes or no), coronary heart disease (yes or no), valvular heart disease (yes or no), reduced renal function (yes or no), prescriptions of ACEIs/ARBs (yes or no), β-blockers (yes or no), and aldosterone antagonists (yes or no).

There were 79 (1.7%), 13 (0.3%), 5 (0.1%), and 4 (0.1%) patients without the value of NT-proBNP, low-density lipoprotein cholesterol, heart rate at discharge, and serum creatinine, respectively. Assuming that they were missing randomly, multiple imputations were utilized to account for missingness. SAS Version 9.4 (SAS Institute, Cary, North Carolina) and R programming language version 4.1.1 (R Foundation for Statistical Computing, Vienna, Austria) were used for all statistical analyses, and a two-sided *P* < 0.05 was considered statistically significant.

## Results

### Baseline Characteristics

In this study, patients who died during hospitalization (*n* = 32), did not attend the follow-up visit (*n* = 9), or without SBP measurement (*n* = 302) were excluded; and 4,564 patients met the inclusion criteria for this study (Supplementary Figure 1). The mean age was 65.3±13.5 years and 1,729 (37.9%) were female; 2,678 (58.7%), 2,647 (58.0%), and 1,669 (36.6%) patients had hypertension, coronary heart disease, and atrial fibrillation, respectively. The average SBP in a stable phase during hospitalization before discharge was 123.2 ± 19.0 mmHg. At discharge, 2,393 (52.4%), 2,720 (59.6%), and 2,918 (63.9%) patients were prescribed ACEIs/ARBs, β-blockers, and aldosterone antagonists, respectively (Table 1).

**Table 1:** Baseline characteristics of patients by SBP groups.

	**Overall** **(*N* = 4,564)**	** <110 mmHg** **(*n* = 1,118)**	**110–119 mmHg** **(*n* = 931)**	**120–129 mmHg** **(*n* = 971)**	**130–139 mmHg** **(*n* = 684)**	**140–149 mmHg** **(*n* = 467)**	**≥150 mmHg** **(*n* = 393)**	***P*** **-value**	***P*** **_trend_-value**
**Demographics**									
Age (yrs), mean ± SD	65.3 ± 13.5	61.3 ± 14.0	64.5 ± 13.6	66.1 ± 12.6	67.7 ± 12.6	68.5 ± 12.1	68.5 ± 13.6	<0.001	<0.001
Female, *n* (%)	1,729 (37.9)	423 (37.8)	324 (34.8)	341 (35.1)	280 (40.9)	174 (37.3)	187 (47.6)	<0.001	0.002
**Socioeconomic status**									
High school education or above, *n* (%)	1,272 (27.9)	330 (29.5)	257 (27.6)	285 (29.4)	184 (26.9)	116 (24.8)	100 (25.4)	0.287	0.042
Marital status: single, *n* (%)	902 (19.8)	192 (17.2)	170 (18.3)	205 (21.1)	131 (19.2)	114 (24.4)	90 (22.9)	0.007	<0.001
**Clinical status**									
Medical history, *n* (%)									
Hypertension	2,678 (58.7)	370 (33.1)	457 (49.1)	612 (63.0)	495 (72.4)	386 (82.7)	358 (91.1)	<0.001	<0.001
Coronary heart disease	2,647 (58.0)	513 (45.9)	515 (55.3)	618 (63.6)	429 (62.7)	305 (65.3)	267 (67.9)	<0.001	<0.001
Atrial fibrillation	1,669 (36.6)	419 (37.5)	365 (39.2)	372 (38.3)	261 (38.2)	151 (32.3)	101 (25.7)	<0.001	<0.001
Diabetes	1,450 (31.8)	272 (24.3)	274 (29.4)	307 (31.6)	226 (33.0)	181 (38.8)	190 (48.3)	<0.001	<0.001
Reduced renal function	1,305 (28.6)	274 (24.5)	223 (24.0)	252 (26.0)	214 (31.3)	153 (32.8)	189 (48.1)	<0.001	<0.001
Anemia	1,060 (23.2)	248 (22.2)	188 (20.2)	190 (19.6)	164 (24.0)	134 (28.7)	136 (34.6)	<0.001	<0.001
Stroke	942 (20.6)	167 (14.9)	171 (18.4)	202 (20.8)	166 (24.3)	135 (28.9)	101 (25.7)	<0.001	<0.001
Chronic obstructive pulmonary disease	899 (19.7)	204 (18.2)	174 (18.7)	213 (21.9)	141 (20.6)	93 (19.9)	74 (18.8)	0.334	0.349
Valvular heart disease	742 (16.3)	265 (23.7)	135 (14.5)	145 (14.9)	98 (14.3)	61 (13.1)	38 (9.7)	<0.001	<0.001
SBP in a stable phase (mmHg), mean ± SD	123.2 ± 19.0	100.3 ± 7.1	114.8 ± 3.0	124.3 ± 2.9	134.3 ± 3.0	144.1 ± 2.9	161.6 ± 10.8	<0.001	<0.001
Heart rate at discharge (beats/min), mean ± SD	73.8 ± 11.0	74.3 ± 11.8	74.2 ± 11.8	74.0 ± 10.2	73.8 ± 10.5	72.6 ± 9.6	72.6 ± 10.3	0.009	<0.001
NYHA class at discharge III/IV, *n* (%)	3474 (76.1)	858 (76.7)	711 (76.4)	737 (75.9)	502 (73.4)	357 (76.4)	309 (78.6)	0.482	0.959
LVEF, mean ± SD	44.8 ± 14.6	40.0 ± 15.0	43.0 ± 14.3	45.0 ± 13.9	48.0 ± 14.0	49.2 ± 13.6	51.1 ± 13.1	<0.001	<0.001
LVEF type, *n* (%)								<0.001	<0.001
HFrEF	1,654 (36.2)	581 (52.0)	371 (39.8)	326 (33.6)	201 (29.4)	102 (21.8)	73 (18.6)		
HFmrEF	1,004 (22.0)	197 (17.6)	216 (23.2)	248 (25.5)	149 (21.8)	109 (23.3)	85 (21.6)		
HFpEF	1,680 (36.8)	285 (25.5)	306 (32.9)	347 (35.7)	307 (44.9)	230 (49.3)	205 (52.2)		
Unmeasured	226 (5.0)	55 (4.9)	38 (4.1)	50 (5.1)	27 (3.9)	26 (5.6)	30 (7.6)		
Decompensation of chronic HF, *n* (%)	3,246 (71.1)	870 (77.8)	679 (72.9)	692 (71.3)	451 (65.9)	300 (64.2)	254 (64.6)	<0.001	<0.001
Body mass index (kg/m^2^), *n* (%)								<0.001	<0.001
<18.5	329 (7.2)	115 (10.3)	70 (7.5)	59 (6.1)	48 (7.0)	22 (4.7)	15 (3.8)		
18.5–23.9	1,893 (41.5)	537 (48.0)	384 (41.2)	408 (42.0)	258 (37.7)	166 (35.5)	140 (35.6)		
≥24	2,138 (46.8)	422 (37.7)	435 (46.7)	460 (47.4)	346 (50.6)	257 (55.0)	218 (55.5)		
Unmeasured	204 (4.5)	44 (3.9)	42 (4.5)	44 (4.5)	32 (4.7)	22 (4.7)	20 (5.1)		
Current smoking, *n* (%)	1,145 (25.1)	287 (25.7)	249 (26.7)	243 (25.0)	168 (24.6)	121 (25.9)	77 (19.6)	0.148	0.055
Central laboratory tests									
NT-proBNP (pg/mL), median (IQR)	1,484 (599, 3,265)	1,819 (875, 3,642)	1,430 (565, 2,947)	1,362 (567, 3,059)	1,365 (528, 2,900)	1,107 (425, 3,039)	1,420 (585, 4,142)	<0.001	<0.001
LDL-C (mmol/L), mean ± SD	2.5 ± 0.9	2.4 ± 0.8	2.5 ± 0.9	2.5 ± 0.9	2.5 ± 0.9	2.7 ± 0.9	2.7 ± 1.0	<0.001	<0.001
**Medication during hospitalization, *n* (%)**									
ACEIs/ARBs	3,069 (67.2)	718 (64.2)	624 (67.0)	640 (65.9)	461 (67.4)	327 (70.0)	299 (76.1)	<0.001	<0.001
β-blockers	3,389 (74.3)	884 (79.1)	712 (76.5)	714 (73.5)	491 (71.8)	328 (70.2)	260 (66.2)	<0.001	<0.001
Aldosterone antagonists	3,802 (83.3)	970 (86.8)	805 (86.5)	814 (83.8)	543 (79.4)	383 (82.0)	287 (73.0)	<0.001	<0.001
Diuretics	4,005 (87.8)	1,009 (90.3)	835 (89.7)	850 (87.5)	582 (85.1)	405 (86.7)	324 (82.4)	<0.001	<0.001
Digoxin	1,556 (34.1)	483 (43.2)	350 (37.6)	308 (31.7)	206 (30.1)	128 (27.4)	81 (20.6)	<0.001	<0.001
Nitrates	2,861 (62.7)	635 (56.8)	568 (61.0)	616 (63.4)	435 (63.6)	326 (69.8)	281 (71.5)	<0.001	<0.001
**Medication at discharge, *n* (%)**									
ACEIs/ARBs	2,393 (52.4)	557 (49.8)	487 (52.3)	493 (50.8)	375 (54.8)	247 (52.9)	234 (59.5)	0.018	0.002
β-blockers	2,720 (59.6)	728 (65.1)	575 (61.8)	563 (58.0)	397 (58.0)	246 (52.7)	211 (53.7)	<0.001	<0.001
Aldosterone antagonists	2,918 (63.9)	783 (70.0)	609 (65.4)	626 (64.5)	416 (60.8)	265 (56.7)	219 (55.7)	<0.001	<0.001
Diuretics	3,164 (69.3)	831 (74.3)	643 (69.1)	675 (69.5)	461 (67.4)	294 (63.0)	260 (66.2)	<0.001	<0.001
Digoxin	1,110 (24.3)	361 (32.3)	248 (26.6)	212 (21.8)	156 (22.8)	81 (17.3)	52 (13.2)	<0.001	<0.001
Nitrates	1,328 (29.1)	273 (24.4)	249 (26.7)	308 (31.7)	196 (28.7)	161 (34.5)	141 (35.9)	<0.001	<0.001
KCCQ score, mean ± SD	43.9 ± 22.8	41.2 ± 22.2	43.7 ± 22.5	45.6 ± 23.1	44.7 ± 23.2	43.7 ± 23.0	46.2 ± 23.6	<0.001	<0.001

*ACEIs, angiotensin converting enzyme inhibitors; ARBs, angiotensin receptor blockers; DBP, diastolic blood pressure; HF, heart failure; HFrEF, heart failure with reduced ejection fraction; HFmrEF, heart failure with mid-range ejection fraction; HFpEF, heart failure with preserved ejection fraction; KCCQ, Kansas City Cardiomyopathy Questionnaire; LVEF, left ventricular ejection fraction; LDL-C, low density lipoprotein cholesterol; NYHA, New York Heart Association; NT-proBNP, N-terminal pro-brain natriuretic peptide; SBP, systolic blood pressure*.

### Systolic Blood Pressure and Clinical Outcomes

**Figure 1:**
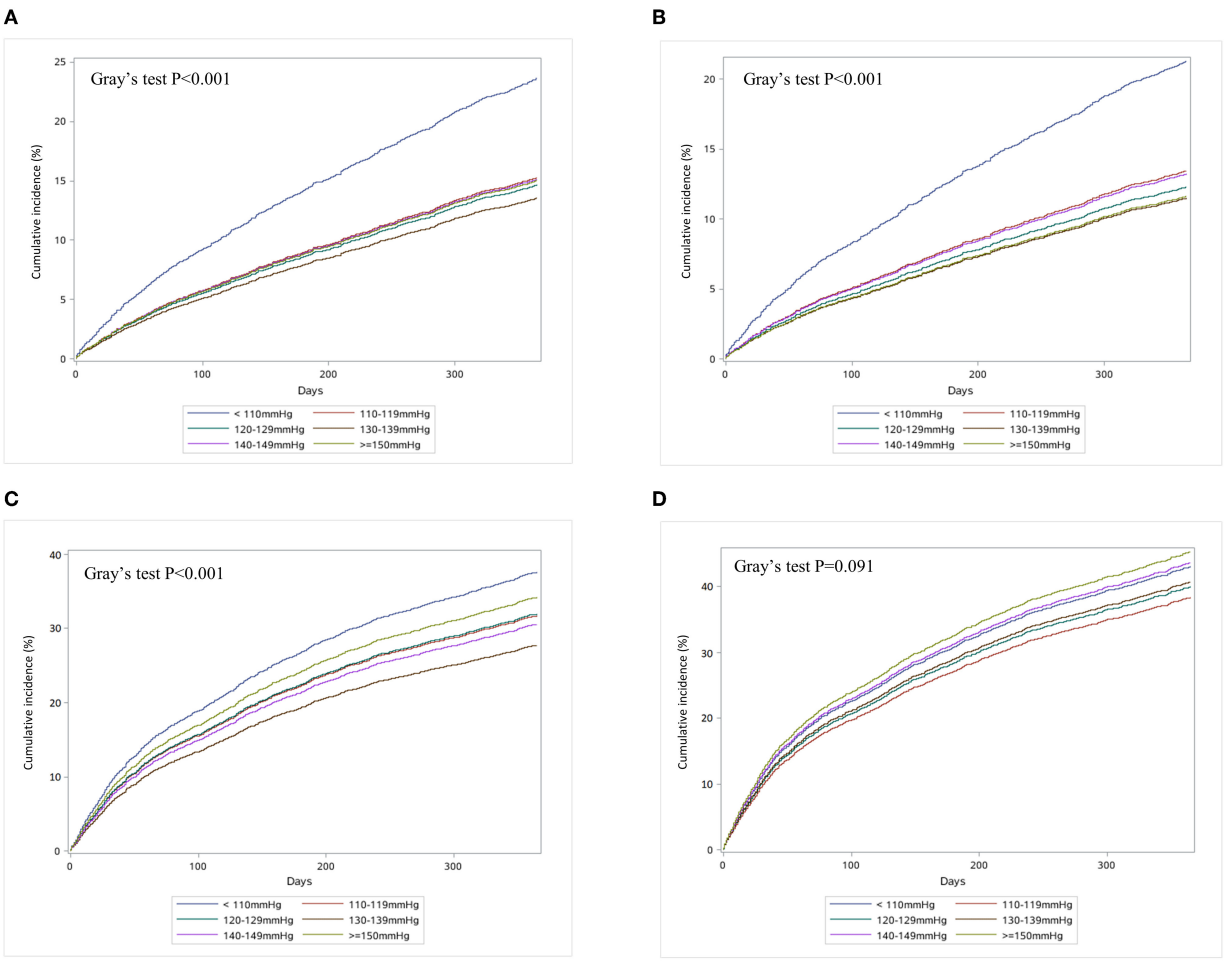
Cumulative incidence curves for all-cause death **(A)**, heart failure readmission **(B)**, cardiovascular death **(C)**, and all-cause hospitalization **(D)** in patients hospitalized for heart failure. This study assessed the association of systolic blood pressure with clinical outcomes in patients hospitalized with heart failure. Patients were categorized into six groups by systolic blood pressure (<110, 110–119, 120–129, 130–139, 140–149, and ≥150 mmHg) for analysis.

During 1-year follow up, 771 (16.9%) deaths, 668 (14.6%) cardiovascular deaths, 1,886 (41.3%) all-cause readmissions, and 1,492 (32.7%) HF readmissions occurred. Of patients with SBP < 110 mmHg, 262 (23.4%) died compared with 93 (13.6%) of those with SBP of 130–139 mmHg. There were 411 (36.8%) patients with SBP < 110 mmHg having HF readmission and 136 (34.6%) patients with SBP ≥ 150 mmHg having HF readmission, compared with 191 (27.9%) of patients with SBP of 130–139 mmHg (Supplementary Table 1). The cumulative incidence curves across SBP groups accounting for the competing risk by the Gray's test were presented in Figure 1.

Compared with SBP of 130–139 mmHg, the adjusted HR for all-cause death was 1.71 (95% CI 1.32–2.20; *P* < 0.001) for SBP < 110 mmHg (Table 2). For HF readmission, patients with SBP < 110 mmHg (adjusted HR, 1.36; 95% CI: 1.14–1.64; *P* = 0.001) and SBP ≥ 150 mmHg (adjusted HR, 1.26; 95% CI: 1.01–1.58; *P* = 0.044) were more likely to have readmission due to HF compared with those with SBP of 130–139 mmHg (Table 2). In sensitivity analysis, the association between SBP and clinical outcomes remained consistent after excluding those who died within 30 days after discharge and those with SBP < 90 mmHg, respectively (Supplementary Tables 2, 3).

**Table 2:** Unadjusted and adjusted risk for outcomes according to SBP groups.

	**All-cause death**		**Heart failure readmission**		**Cardiovascular death**		**All-cause readmission**	
	**HR (95% CI)**	***P*** **-value**	**HR (95% CI)**	***P*** **-value**	**HR (95% CI)**	***P*** **-value**	**HR (95% CI)**	***P*** **-value**
<110 mmHg	1.96 (1.54, 2.49)	<0.001	1.45 (1.22, 1.72)	<0.001	1.96 (1.52, 2.53)	<0.001	1.08 (0.93, 1.25)	0.324
110–119 mmHg	1.15 (0.89, 1.50)	0.287	1.17 (0.98, 1.41)	0.080	1.18 (0.89, 1.57)	0.242	0.93 (0.79, 1.08)	0.322
120–129 mmHg	1.10 (0.85, 1.43)	0.463	1.19 (0.99, 1.42)	0.061	1.07 (0.81, 1.43)	0.618	0.98 (0.84, 1.14)	0.760
130–139 mmHg	1.00		1.00		1.00		1.00	
140–149 mmHg	1.17 (0.86, 1.59)	0.329	1.12 (0.91, 1.39)	0.291	1.16 (0.83, 1.62)	0.372	1.10 (0.92, 1.31)	0.310
≥150 mmHg	1.15 (0.83, 1.59)	0.403	1.29 (1.04, 1.60)	0.022	1.01 (0.71, 1.45)	0.948	1.15 (0.96, 1.39)	0.132
**Adjusted for demographic, socioeconomic, clinical characteristics, treatment, and self-reported health status**								
<110 mmHg	1.71 (1.32, 2.20)	<0.001	1.36 (1.14, 1.64)	0.001	1.66 (1.27, 2.18)	<0.001	1.11 (0.95, 1.30)	0.182
110–119 mmHg	1.17 (0.90, 1.53)	0.248	1.17 (0.97, 1.41)	0.103	1.15 (0.86, 1.52)	0.348	0.95 (0.81, 1.11)	0.530
120–129 mmHg	1.07 (0.82, 1.39)	0.619	1.16 (0.96, 1.39)	0.120	1.02 (0.76, 1.36)	0.896	0.97 (0.83, 1.13)	0.689
130–139 mmHg	1.00		1.00		1.00		1.00	
140–149 mmHg	1.13 (0.83, 1.55)	0.433	1.16 (0.93, 1.44)	0.192	1.16 (0.83, 1.63)	0.374	1.08 (0.91, 1.30)	0.381
≥150 mmHg	0.95 (0.68, 1.33)	0.784	1.26 (1.01, 1.58)	0.044	0.86 (0.59, 1.25)	0.432	1.11 (0.91, 1.34)	0.301

*CI, confidence interval; HR, hazard ratio; SBP, systolic blood pressure*.

**Figure 2:**
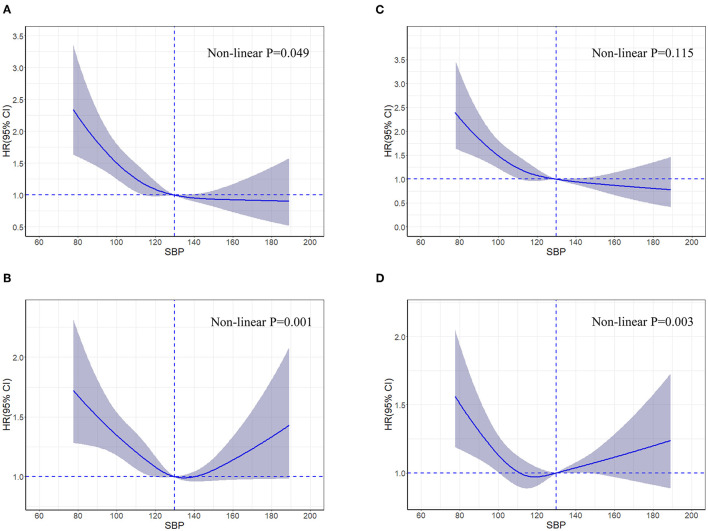
Restricted cubic spline curves for an association between systolic blood pressure and all-cause death **(A)**, heart failure readmission **(B)**, cardiovascular death **(C)**, and all-cause hospitalization **(D)** in patients hospitalized for heart failure. Hazard ratios and 95% confidence intervals for clinical outcomes by systolic blood pressure groups in 4,564 patients hospitalized for heart failure according to restricted cubic spline regression models using four knots at systolic blood pressures of 95, 115, 129, and 157 mmHg. SBP of 130 mmHg was applied as the reference. Solid blue lines indicate hazard ratios, and shaded areas indicate 95% confidence intervals. CI, confidence interval; HR, hazard ratio; SBP, systolic blood pressure.

In the restricted cubic spline analysis, the risk of all-cause death increased significantly only at lower SBP (Figure 2); while a J-shaped association was observed between SBP and HF readmission (*P* = 0.001), and the nadir of risk occurred when the SBP was around 130 mmHg with the risk increasing below or above that cutoff (Figure 2).

### Subgroup Analyses

**Figure 3:**
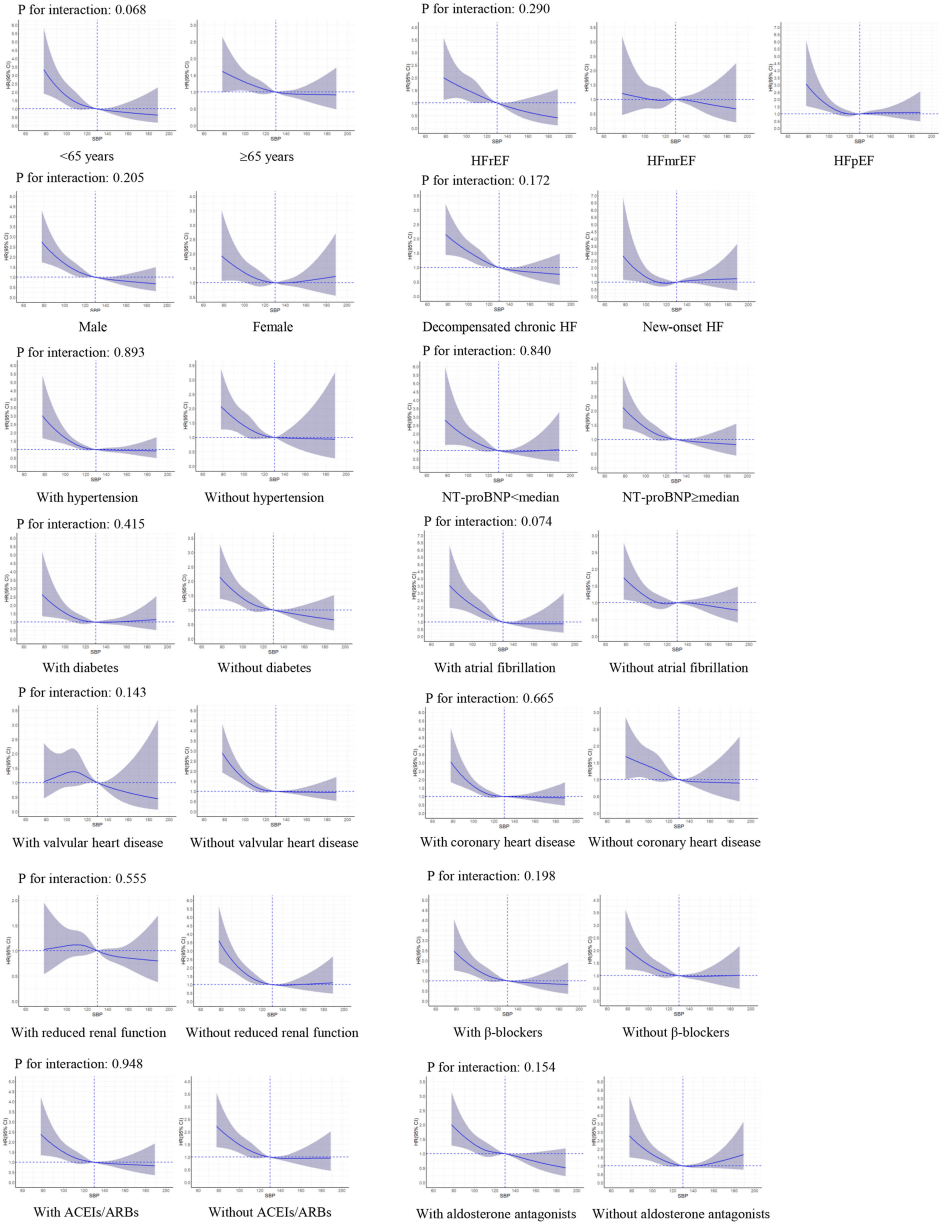
Restricted cubic spline curves for an association between systolic blood pressure and all-cause death in subgroups. SBP of 130 mmHg was applied as the reference. ACEIs, angiotensin-converting enzyme inhibitors; ARBs, angiotensin II receptor blockers; CI, confidence interval; HR, hazard ratio; HF, heart failure; HFrEF, heart failure with reduced ejection fraction; HFmrEF, heart failure with mid-range ejection fraction; HFpEF, heart failure with preserved ejection fraction; NT-proBNP, N-terminal pro-brain natriuretic peptide; SBP, systolic blood pressure.

**Figure 4:**
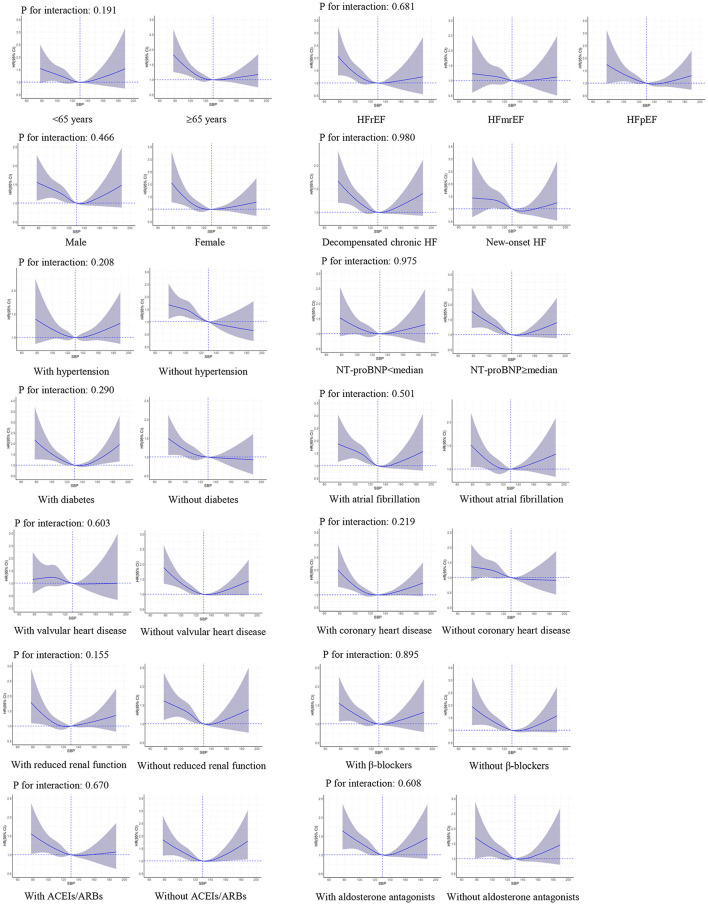
Restricted cubic spline curves for an association between systolic blood pressure and HF readmission in subgroups. SBP of 130 mmHg was applied as the reference. ACEIs, angiotensin-converting enzyme inhibitors; ARBs, angiotensin II receptor blockers; CI, confidence interval; HR, hazard ratio; HF, heart failure; HFrEF, heart failure with reduced ejection fraction; HFmrEF, heart failure with mid-range ejection fraction; HFpEF, heart failure with preserved ejection fraction; NT-proBNP, N-terminal pro-brain natriuretic peptide; SBP, systolic blood pressure.

There were no significant interactions of SBP level with LVEF (*P*-value for interaction: 0.290 for all-cause death and 0.681 for HF readmission, respectively). The interactions were not significant in other important subgroups, including age, sex, HF type, hypertension, diabetes, coronary heart disease, prescriptions of ACEIs/ARBs, β-blockers, and aldosterone antagonists (Figures 3, 4).

## Discussion

Findings from this study demonstrate that lower SBP in a stable phase during hospitalization was associated with an increased risk of 1-year death in patients hospitalized for HF, and our study for the first time observed a J-curve association between SBP in a stable phase during hospitalization and 1-year HF readmission. The associations remained consistent in various clinically important subgroups, including the subgroups of age, sex, LVEF, HF type, hypertension, and coronary heart disease; the consistency was not affected when using important medications for HF with BP-lowering effects.

Our study expands the literature by providing an insight into the association of SBP and death among a whole spectrum of HF with more accurate BP measurement. In our study, we measured BP in a stable phase during hospitalization before discharge according to the standard approach; i.e., using the unified BP monitor and measuring BP at least twice to get the average. This approach maximumly reduced the measurement bias, and we could therefore obtain more accurate SBP values than previous studies which only collected SBP values from routine clinical measurement at discharge (12–16). Our study demonstrated patients with SBP < 110 mmHg had a 1.7-fold higher risk of death than those with SBP of 130–139 mmHg. Similar to our findings, several other studies found an inverse association of SBP at discharge with the risk of death in patients hospitalized for HF (12–14, 20). However, these studies only included patients with HFrEF or HFpEF (12–14, 20), while patients with HFmrEF were usually overlooked and less well-studied. In our study, all three types of patients were included and we did not observe heterogeneity in the associations between SBP and death across LVEF levels. In the sensitivity analysis, we excluded the patients with SBP < 90 mmHg, who were more likely to have worse clinical status and greater adverse outcomes (25), and the association remained consistent. Accordingly, our findings suggest that clinicians should be cautious about the lowering of SBP to a low level during hospitalization for patients with HF.

Our data suggest that the association between SBP < 110 mmHg and increased risk may be causal rather than due to reverse causality. It is possible that patients with lower SBP had more progressive systolic dysfunction and the resultant neurohormonal activation may result in a higher risk of death (26); however, the effects of SBP on death were homogeneous in patients with HF with different LVEF levels. In addition, lower BP might cause coronary hypoperfusion and result in increased deaths among patients with the compromised coronary flow (27, 28); nevertheless, the associations were consistent in patients with and without coronary heart disease. Another possible explanation could be related to the treatment patterns. Because patients with lower SBP tended to be undertreated with HF therapy, prior studies left open the possibility that suboptimal treatment could contribute to the poor outcomes of these patients (29); however, in this study, among patients with SBP <110 mmHg, most of them have been treated with proved medications during hospitalization and at discharge. The use of β-blockers and aldosterone antagonists during hospitalization and at discharge was even higher in patients with SBP <110 mmHg than other SBP groups. Besides, the association between SBP and death was significant no matter whether patients received these medications or not. Moreover, we excluded the patients who died within 30 days after discharge because of their greater burden of comorbidities and higher illness severities, and the results remained the same (30). Thus, this study indicates that lower SBP may not be a marker of more advanced disease or worse health status, which suggests that SBP *per se* may be directly associated with the increased risks. And the randomized clinical trials to evaluate the optimal SBP goals in HF patients are warranted.

This study is the first to reveal a J-curve association between SBP in a stable phase during hospitalization and 1-year HF readmission in patients hospitalized for HF with the nadir SBP around 130 mmHg. Some previous studies have examined the association between discharge SBP and HF readmission, but they did not evaluate whether the non-linear association existed (14, 20). The association between SBP and HF readmission in this study agreed with the results from a previous analysis by Lee et al. which described a non-linear association between on-treatment SBP and long-term HF readmission in a Korean acute heart failure cohort, with the lowest risk at an SBP of 144 mmHg (19). In our study, among patients with SBP ≥ 130 mmHg, more than three quarters had a medical history of hypertension; and in those with SBP ≥ 150 mmHg, more than 90% had hypertension. Persistent high SBP could increase the left ventricular afterload and peripheral vascular resistance, leading to a high possibility of the clinical deterioration (31). As a result, it is possible that HF patients with higher SBP are more likely to have readmission due to HF aggravation (19). More investigations are needed to confirm the findings and further elucidate the underlying mechanism.

This study should be considered in the context of limitations. Although we performed multivariable analyses accounting for extensive baseline characteristics, unmeasured and residual confounders could exist, which is inevitable for the observational studies. We had limited data on post-discharge SBP, and SBP crossover during follow-up may attenuate between-group differences in the outcomes. As this study was based on patients hospitalized for HF, the results could not be generalizable to other HF populations, especially ambulatory HF patients, because the clinical characteristics of patients in these two settings are different.

In conclusion, among patients hospitalized for HF, lower SBP in a stable phase during hospitalization portends an increased risk of 1-year death, and a J-curve association has been observed between SBP and 1-year HF readmission with the nadir SBP around 130 mmHg. The associations remained consistent across various subgroups of clinical importance. Our observation could provide more evidence for future recommendations on BP management among patients with HF.

## Data Availability Statement

The datasets presented in this article are not readily available because, the China PEACE 5p-HF Study is a national program, and as the government policy stipulates, it is not permissible for the researchers to make the raw data publicly available at this time. Requests to access the datasets should be directed to JLi, jing.li@fwoxford.org.

## Ethics Statement

The studies involving human participants were reviewed and approved by the Central Ethics Committee at Fuwai Hospital and Local Internal Ethics Committees at Study Hospitals. The patients/participants provided their written informed consent to participate in this study.

## Author Contributions

JLi, JLiu, and XH designed the study. XH, XB, and SH designed the statistical methods and analyzed the data. XH drafted the manuscript. LZ, BW, FM, AT, TY, and YL revised the manuscript for important intellectual content. All the authors participated in the interpretation of the data and approved the final version of the manuscript.

## Funding

This work was supported by the National Key Research and Development Program (2018YFC1312400, 2018YFC1312401, and 2018YFC1312404) from the Ministry of Science and Technology of China, the CAMS Innovation Fund for Medical Science (2021-1-I2M-009), and the National Key Technology R&D Program (2015BAI12B02) from the Ministry of Science and Technology of China.

## Conflict of Interest

JLi reported receiving research grants, through Fuwai Hospital, from the Chinese government and Chinese Academy of Medical Sciences for work to improve the management of hypertension and blood lipids and to improve patient outcomes of cardiovascular disease and COVID-19; receiving research agreements, through the National Center for Cardiovascular Diseases and Fuwai Hospital, from Amgen for a multicenter clinical trial assessing the efficacy and safety of omecamtiv mecarbil and for dyslipidemic patient registration; receiving a research agreement, through the Fuwai Hospital, from Sanofi for a multicenter clinical trial on the effects of sotagliflozin; receiving a research agreement, through the Fuwai Hospital, with the University of Oxford for a multicenter clinical trial of empagliflozin; receiving a research agreement, through the National Center for Cardiovascular Diseases, from AstraZeneca for clinical research methods training outside the submitted work; and receiving a research agreement, through the National Center for Cardiovascular Diseases, from Lilly for physician training outside the submitted work. The remaining authors declare that the research was conducted in the absence of any commercial or financial relationships that could be construed as a potential conflict of interest.

## Publisher's Note

All claims expressed in this article are solely those of the authors and do not necessarily represent those of their affiliated organizations, or those of the publisher, the editors and the reviewers. Any product that may be evaluated in this article, or claim that may be made by its manufacturer, is not guaranteed or endorsed by the publisher.
