# Supplementary material for: Systolic Blood Pressure and 1-Year Clinical Outcomes in Patients Hospitalized for Heart Failure
Source: Front Cardiovasc Med. 2022 Apr 25;9:877293. doi: 10.3389/fcvm.2022.877293 (PMC9081363; doi:10.3389/fcvm.2022.877293)
Supplement: Supplementary file 1 [file Data_Sheet_1.docx]

**Systolic blood pressure and one-year clinical outcomes in patients hospitalized for heart failure**

Xinghe Huang, PhD^1,2,3^, Jiamin Liu, MD^2*^, Lihua Zhang, MD, PhD^2^, Bin Wang, MD, PhD^2^, Xueke Bai, MS^2^, Shuang Hu, PhD^2^, Fengyu Miao, PhD^2^, Aoxi Tian, MS^2^, Tingxuan Yang, BS^2^, Yan Li, MD^2^, Jing Li, MD, PhD^1,2*^

**Author Affiliations**

^1^ Fuwai Hospital, Chinese Academy of Medical Sciences, Shenzhen, People's Republic of China

^2^ National Clinical Research Center for Cardiovascular Diseases, Fuwai Hospital, Chinese Academy of Medical Sciences and Peking Union Medical College, National Center for Cardiovascular Diseases, Beijing, People's Republic of China

^3^ School of Nursing, Chinese Academy of Medical Sciences and Peking Union Medical College, Beijing, People's Republic of China

*** Corresponding author:**

Professor Jing Li, Building C, National Center for Cardiovascular Diseases, Beijing, 102308, Shilongxi Road, Mentougou District, Beijing, P. R. China; Tel: +86 10 6086 6077; Email: [jing.li@fwoxford.org](mailto:jing.li@fwoxford.org)

Professor Jiamin Liu, Building C, National Center for Cardiovascular Diseases, Beijing, 102308, Shilongxi Road, Mentougou District, Beijing, P. R. China; Tel: +86 10 6086 6896; Email: [jiamin.liu@fwoxford.org](mailto:jing.li@fwoxford.org)

**Supplementary Appendix**

**Systolic blood pressure and one-year clinical outcomes in patients hospitalized for heart failure**

[Supplementary Tables 2](#_Toc90283247)

[Table S1 Event rates of clinical outcomes according to SBP groups 2](#_Toc90283248)

[Table S2 Unadjusted and adjusted risk for outcomes according to SBP groups after excluding those who died within 30 days after discharge 3](#_Toc90283249)

[Table S3 Unadjusted and adjusted risk for outcomes according to SBP groups after excluding those with SBP <90 mmHg 5](#_Toc90283250)

[Supplementary Figures 7](#_Toc90283251)

[Figure S1 Flowchart of patient selection 7](#_Toc90283252)

[Full list of names and principal investigators of sites 8](#_Toc90283253)

# Supplementary Tables

## Table S1 Event rates of clinical outcomes according to SBP groups

|  | All-cause death (%) | Cardiovascular death (%) | All-cause readmission (%) | Heart failure readmission (%) |
| --- | --- | --- | --- | --- |
| <110 mmHg | 23.4 | 21.1 | 42.0 | 36.8 |
| 110-119 mmHg | 15.3 | 13.4 | 38.7 | 31.9 |
| 120-129 mmHg | 14.7 | 12.4 | 40.3 | 32.2 |
| 130-139 mmHg | 13.6 | 11.6 | 41.2 | 27.9 |
| 140-149 mmHg | 15.2 | 13.3 | 43.7 | 30.8 |
| ≥150 mmHg | 15.3 | 11.7 | 45.6 | 34.6 |

## Table S2 Unadjusted and adjusted risk for outcomes according to SBP groups after excluding those who died within 30 days after discharge

|  | **All-cause death** | |  | **Heart failure readmission** | |  | **Cardiovascular death** | |  | **All-cause readmission** | |
| --- | --- | --- | --- | --- | --- | --- | --- | --- | --- | --- | --- |
|  | **HR (95% CI)** | **P Value** |  | **HR (95% CI)** | **P Value** |  | **HR (95% CI)** | **P Value** |  | **HR (95% CI)** | **P Value** |
| Unadjusted | | | | | | | | | | | |
| <110 mmHg | 1.75 (1.35, 2.27) | <0.001 |  | 1.44 (1.21, 1.71) | <0.001 |  | 1.80 (1.37, 2.38) | <0.001 |  | 1.06 (0.91, 1.24) | 0.435 |
| 110-119 mmHg | 1.12 (0.84, 1.48) | 0.446 |  | 1.18 (0.98, 1.41) | 0.079 |  | 1.16 (0.86, 1.58) | 0.322 |  | 0.94 (0.80, 1.09) | 0.403 |
| 120-129 mmHg | 1.13 (0.86, 1.49) | 0.390 |  | 1.18 (0.99, 1.42) | 0.067 |  | 1.12 (0.83, 1.51) | 0.457 |  | 1.00 (0.85, 1.16) | 0.959 |
| 130-139 mmHg | 1.00 |  |  | 1.00 |  |  | 1.00 |  |  | 1.00 |  |
| 140-149 mmHg | 1.16 (0.83, 1.61) | 0.383 |  | 1.13 (0.91, 1.40) | 0.279 |  | 1.16 (0.81, 1.66) | 0.412 |  | 1.10 (0.92, 1.32) | 0.288 |
| ≥150 mmHg | 1.11 (0.78, 1.58) | 0.551 |  | 1.30 (1.04, 1.62) | 0.020 |  | 0.96 (0.65, 1.42) | 0.820 |  | 1.17 (0.97, 1.41) | 0.107 |
| Adjusted for demographic, socioeconomic, clinical characteristics, treatment and self-reported health status | | | | | | | | | | | |
| <110 mmHg | 1.50 (1.14, 1.97) | 0.004 |  | 1.36 (1.13, 1.63) | 0.001 |  | 1.54 (1.15, 2.05) | 0.004 |  | 1.10 (0.94, 1.29) | 0.244 |
| 110-119 mmHg | 1.11 (0.83, 1.48) | 0.476 |  | 1.18 (0.97, 1.42) | 0.091 |  | 1.14 (0.84, 1.55) | 0.409 |  | 0.96 (0.82, 1.13) | 0.618 |
| 120-129 mmHg | 1.08 (0.81, 1.42) | 0.612 |  | 1.16 (0.96, 1.39) | 0.125 |  | 1.05 (0.77, 1.43) | 0.742 |  | 0.99 (0.85, 1.15) | 0.866 |
| 130-139 mmHg | 1.00 |  |  | 1.00 |  |  | 1.00 |  |  | 1.00 |  |
| 140-149 mmHg | 1.13 (0.81, 1.57) | 0.488 |  | 1.16 (0.93, 1.45) | 0.174 |  | 1.15 (0.81, 1.65) | 0.439 |  | 1.10 (0.91, 1.31) | 0.331 |
| ≥150 mmHg | 0.93 (0.65, 1.33) | 0.696 |  | 1.28 (1.02, 1.60) | 0.035 |  | 0.80 (0.53, 1.20) | 0.284 |  | 1.13 (0.93, 1.37) | 0.213 |

Abbreviations: CI: confidence interval; HR: hazard ratio; SBP: systolic blood pressure

## Table S3 Unadjusted and adjusted risk for outcomes according to SBP groups after excluding those with SBP <90 mmHg

|  | **All-cause death** | |  | **Heart failure readmission** | |  | **Cardiovascular death** | |  | **All-cause readmission** | |
| --- | --- | --- | --- | --- | --- | --- | --- | --- | --- | --- | --- |
|  | **HR (95% CI)** | **P Value** |  | **HR (95% CI)** | **P Value** |  | **HR (95% CI)** | **P Value** |  | **HR (95% CI)** | **P Value** |
| Unadjusted | | | | | | | | | | | |
| <110 mmHg | 1.88 (1.47, 2.40) | <0.001 |  | 1.44 (1.21, 1.72) | <0.001 |  | 1.87 (1.45, 2.42) | <0.001 |  | 1.06 (0.91, 1.23) | 0.456 |
| 110-119 mmHg | 1.15 (0.89, 1.50) | 0.291 |  | 1.17 (0.98, 1.41) | 0.080 |  | 1.18 (0.89, 1.57) | 0.242 |  | 0.93 (0.79, 1.08) | 0.321 |
| 120-129 mmHg | 1.10 (0.85, 1.43) | 0.473 |  | 1.19 (0.99, 1.42) | 0.061 |  | 1.07 (0.81, 1.43) | 0.618 |  | 0.98 (0.84, 1.14) | 0.760 |
| 130-139 mmHg | 1.00 |  |  | 1.00 |  |  | 1.00 |  |  | 1.00 |  |
| 140-149 mmHg | 1.16 (0.85, 1.59) | 0.342 |  | 1.12 (0.91, 1.39) | 0.291 |  | 1.16 (0.83, 1.62) | 0.372 |  | 1.10 (0.92, 1.31) | 0.310 |
| ≥150 mmHg | 1.15 (0.83, 1.59) | 0.409 |  | 1.29 (1.04, 1.60) | 0.022 |  | 1.01 (0.71, 1.45) | 0.948 |  | 1.15 (0.96, 1.39) | 0.132 |
| Adjusted for demographic, socioeconomic, clinical characteristics, treatment and self-reported health status | | | | | | | | | | | |
| <110 mmHg | 1.67 (1.29, 2.16) | <0.001 |  | 1.37 (1.14, 1.65) | 0.001 |  | 1.63 (1.24, 2.15) | <0.001 |  | 1.09 (0.93, 1.28) | 0.271 |
| 110-119 mmHg | 1.17 (0.89, 1.53) | 0.257 |  | 1.17 (0.97, 1.41) | 0.096 |  | 1.15 (0.87, 1.53) | 0.330 |  | 0.95 (0.81, 1.11) | 0.533 |
| 120-129 mmHg | 1.07 (0.82, 1.39) | 0.632 |  | 1.16 (0.96, 1.39) | 0.119 |  | 1.02 (0.76, 1.37) | 0.883 |  | 0.97 (0.83, 1.13) | 0.692 |
| 130-139 mmHg | 1.00 |  |  | 1.00 |  |  | 1.00 |  |  | 1.00 |  |
| 140-149 mmHg | 1.13 (0.83, 1.55) | 0.434 |  | 1.16 (0.93, 1.44) | 0.194 |  | 1.17 (0.84, 1.63) | 0.363 |  | 1.09 (0.91, 1.30) | 0.370 |
| ≥150 mmHg | 0.95 (0.68, 1.33) | 0.778 |  | 1.26 (1.00, 1.57) | 0.047 |  | 0.86 (0.59, 1.25) | 0.424 |  | 1.10 (0.91, 1.34) | 0.311 |

Abbreviations: CI: confidence interval; HR: hazard ratio; SBP: systolic blood pressure

# Supplementary Figures

##
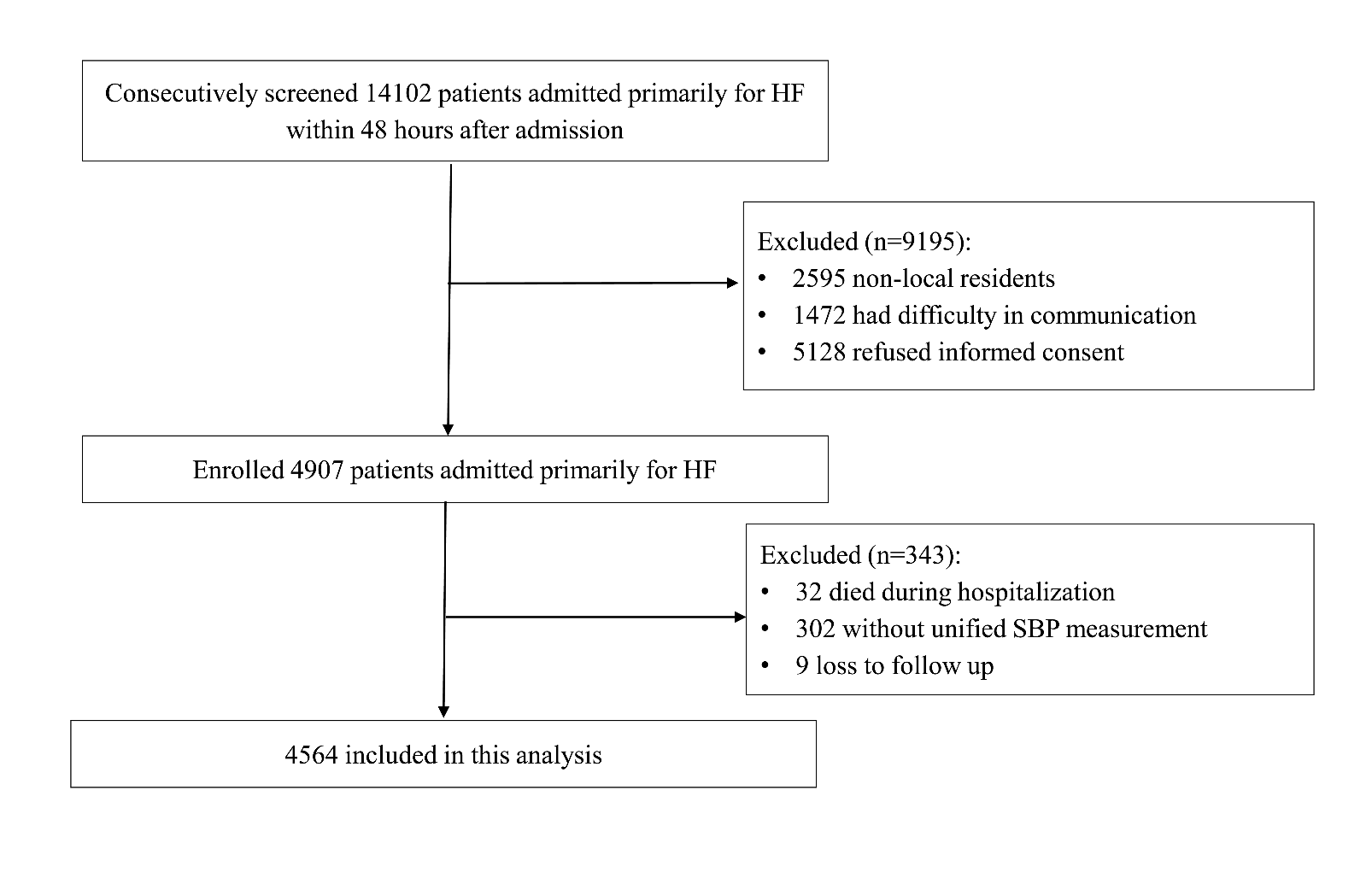
Figure S1 Flowchart of patient selection

# Full list of names and principal investigators of sites

| Hospital | Province/ Municipality | PI |
| --- | --- | --- |
| The Center Hospital of Maanshan | Anhui | Qian Jun |
| Beijing Liangxiang Hospital | Beijing | Fang Xuehua |
| Beijing Chaoyang Hospital | Beijing | Wei Yu |
| Fuwai Hospital, Chinese Academy of Medical Sciences, Peking Union Medical College | Beijing | Zhang Yuhui |
| The First Affiliated Hospital of Chongqing Medical University | Chongqing | Luo Suxin |
| Xiamen Cardiovascular Hospital Xiamen University | Fujian | Dai Cuilian |
| Guangdong Provincial People's Hospital | Guangdong | Chen Jiyan |
| Pan Yu Branch of the Second Affiliated Hospital Of Guangzhou Medical University | Guangdong | Cui Yongsheng |
| The People's Hospital of Guangxi Zhuang Autonomous Region | Guangxi | Wu Guangwei |
| The First Affiliated Hospital of Hebei North University | Hebei | Li Fangjiang |
| Luoyang Dongfang Hospital | Henan | Zhang Ge |
| Puyang Oilfield General Hospital | Henan | Wang Hengliang |
| Qinyang People's Hospital | Henan | Ma Xiaowen |
| Xinxiang Central Hospital | Henan | Wang Zhifang |
| The Affiliated Hospital of Xuzhou Medical University | Henan | Li Dongye |
| The first Affiliated Hospital of Zhengzhou University | Henan | Fu Xin |
| Brain Hospital of Hunan Province | Hunan | Xing Zuzhong |
| The First Affiliated Hospital of University of South China | Hunan | Tang Huifang |
| Inner Mongolia International Mongolian Hospital | Inner Mongolia | Mo Rigentu |
| Hulunbeir People's Hospital | Inner Mongolia | Cui Zhonghua |
| Baogang Hospital | Inner Mongolia | Li Yongdong |
| The Second Affiliated Hospital of Baotou Medical college | Inner Mongolia | Sun Gang |
| The Affiliated Hospital of Inner Mongolia Medical University | Inner Mongolia | Liu Juan |
| Inner Mongolia People's Hospital | Inner Mongolia | Han Yajun |
| Inner Mongolia Hospital of Traditional Chinese Medicine | Inner Mongolia | Su He |
| The Second Affiliated Hospital of Xuzhou Medical University | Jiangsu | Wu Weiheng |
| The First Hospital of Jilin University | Jilin | Zheng Yang |
| China-Japan Union Hospital of Jilin University | Jilin | Yang Ping |
| Anshan Changda Hospital | Liaoning | Jin Xiang |
| Benxi Jinshan Hospital | Liaoning | Xiu Guoquan |
| Affiliated Zhongshan Hospital of Dalian University | Liaoning | Yu Qin |
| Shenyang the Fourth Hospital of People | Liaoning | Li Yinjun |
| Shenyang First People's Hospital | Liaoning | Xu Jian |
| Central Hospital of Shenyang Sujiatun District | Liaoning | Che Hang |
| Xinmin People's Hospital | Liaoning | Qi Liwei |
| Qinghai Cardiovascular and Cerebrovascular Hospital | Qinghai | Ma Xiaofeng |
| Affiliated Hospital of Jining Medical University | Shandong | Zhang Jinguo |
| Qingdao Fuwai Cardiovascular Hospital | Shandong | Jiang Xianyan |
| Shanxi Fenyang Hospital | Shanxi | Li Li |
| Quwo County People's Hospital | Shanxi | Wu Zhenlin |
| Second Hospital of Shanxi Medical University | Shanxi | Yang Huiyu |
| Taiyuan City Central Hospital | Shanxi | Chen Xiaoping |
| The First Affiliated Hospital of Xi'an Jiaotong University | Shanxi | Ren Jie |
| XIAN NO.1 Hospital | Shanxi | Ji Yuqiang |
| Nanchong Central Hospital | Sichuan | Wang Haoyu |
| No.363 Hospital | Sichuan | Ning Liang |
| Hospital of Chendu Office of People's Government of Tibetan Autonomous Region | Sichuan | Zhou Yao |
| First Affiliated Hospital of Kunming Medical University | Yunnan | Cai Hongyan |
| The Affiliated Yueqing Hospital of Wenzhou Medical University | Zhejiang | Yu Xudong |
| Ningbo First Hospital | Zhejiang | Cui Hanbin |
| Taizhou Hospital of Zhejiang Province | Zhejiang | Jiang Jianjun |
| Zhejiang Hospital | Zhejiang | Yan Jing |
